# Cross-Presentation and Activation of CD8+ T Cells: The Role of Pannexin-1 in Dendritic Cells

**DOI:** 10.3390/ijms27125559

**Published:** 2026-06-19

**Authors:** Francisco Bravo, Paulina Troncoso, Javier Mena, Catalina Bascuñan, Nayiberg Varas, Daniela Sauma, Claudio Acuña-Castillo, Carlos Barrera-Avalos

**Affiliations:** 1 Centro de Biotecnología Acuícola, Facultad de Química y Biología, Universidad de Santiago de Chile, Santiago 9160000, Chile; francisco.bravo.c@usach.cl (F.B.); javier.mena@usach.cl (J.M.); nayiberg.varas@usach.cl (N.V.); 2 Departamento de Biología, Facultad de Ciencias, Universidad de Chile, Santiago 9160000, Chile; paulina.troncoso.c@usach.cl (P.T.); catalinabascunanr@gmail.com (C.B.); dsauma@uchile.cl (D.S.); 3 Departamento de Biología, Facultad de Química y Biología, Universidad de Santiago de Chile, Santiago 9160000, Chile

**Keywords:** cross-presentation, Pannexin-1, cytosolic pathway, dendritic cells

## Abstract

Cross-presentation of exogenous antigens by dendritic cells (DCs) relies on the cytosolic pathway, enabling proteasomal processing and subsequent loading of antigenic peptides onto major histocompatibility complex class I (MHC-I) molecules. Although this pathway is central to CD8^+^ T-cell activation, the molecular mechanisms that regulate intracellular antigen processing and redistribution during cross-presentation remain incompletely defined. In this study, we investigated the contribution of the large-pore channel Pannexin-1 (Panx1) to antigen handling during cross-presentation. Using confocal microscopy and quantitative image analysis in granulocyte–macrophage colony-stimulating factor/interleukin-4 (GM-CSF/IL-4)-derived inflammatory bone marrow-derived dendritic cell (BMDC)-like cellsexposed to ovalbumin (OVA)–Alexa Fluor 488, we observed time-dependent changes in intracellular antigen distribution that were altered upon pharmacological inhibition of Panx1 with the blocking peptide 10Panx1. In parallel, functional assays revealed that Panx1 inhibition significantly reduced SIINFEKL peptide-dependentactivation of B3Z CD8^+^ T-cell hybridomas following pulsing with full-length OVA. Similar effects were observed in the cross-presentation-competent MUTU1940 dendritic cell line. Importantly, Panx1 inhibition did not significantly affect dendritic-cell viability or LPS-induced activation under the experimental conditions tested. In contrast, pharmacological inhibition or genetic deficiency of P2X7 receptor (P2X7) did not produce comparable reductions in cross-presentation, and combined inhibition did not result in additive effects under the experimental conditions tested. Together, these findings provide functional evidence supporting a role for Panx1 in regulating intracellular antigen redistribution associated with cross-presentation. While not establishing direct genetic causality, our data identify Panx1 as a modulatory component influencing antigen-processing events that culminate in CD8^+^ T-cell activation, thereby expanding the current framework of intracellular antigen-processing mechanisms involved in dendritic-cell-mediated cross-presentation.

## 1. Introduction

Cross-presentation of antigens is an essential process for the activation of CD8^+^ cytotoxic T lymphocytes (CTLs), enabling effective immune responses against infected or malignant cells [1]. This mechanism is primarily mediated by dendritic cells (DCs), which capture exogenous antigens and process them for presentation on major histocompatibility complex class I (MHC-I) molecules, thereby promoting CTL activation [2]. Despite its central importance, the intracellular mechanisms that allow internalized antigens to gain access to the cytosolic processing pathway remain incompletely defined and continue to be actively investigated [3].

The cytosolic pathway of cross-presentation, which involves proteasomal degradation of antigens prior to loading onto MHC-I molecules, is considered the predominant route in many experimental systems [4]. This pathway requires a mechanism capable of enabling antigenic material to move from endocytic or phagosomal compartments into a cytosol-accessible pool. Early models proposed that components of the endoplasmic reticulum-associated degradation (ERAD) machinery, particularly the Sec61 translocon, could mediate this export step [5]. However, subsequent studies challenged this hypothesis: inhibition of Sec61 reduced cross-presentation without consistently impairing antigen access to the cytosol [6], and RNA interference targeting Sec61 did not significantly alter cytosolic antigen delivery [7]. Similar observations were reported for other ERAD-associated components, including Derlin-1 and Hrd1, which also failed to demonstrate a direct and universal role in antigen translocation [8,9,10]. More recently, alternative mechanisms have been proposed, including controlled permeabilization of antigen-containing compartments mediated by pore-forming proteins. In this context, Perforin-2 (MPEG1) has emerged as a factor capable of facilitating antigen access to the cytosolic pathway [11]. Together, these findings support the concept that cytosolic access during cross-presentation is mediated by multiple, potentially complementary mechanisms or other dedicated transporters.

The capacity for cross-presentation is not homogeneous across dendritic-cell populations. Conventional type 1 dendritic cells (cDC1s) are particularly efficient at cross-presentation and CD8^+^ T-cell priming, whereas Conventional type 2 dendritic cells (cDC2) populations are more closely associated with CD4^+^ T-cell activation and helper responses [12,13]. In experimental settings, bone marrow-derived dendritic cells (BMDCs) generated in the presence of granulocyte–macrophage colony-stimulating factor (GM-CSF)and interleukin-4 (IL-4)represent heterogeneous inflammatory BMDC-like populations rather than ontogenically defined cDC subsets. Accordingly, these cells cannot be assumed to correspond to bona fide cDC2 populations in vivo, and mechanisms identified in this system must be interpreted within this context (see also [14,15]). Complementary use of cell lines such as MUTU1940, which display cDC1-like features and robust cross-presentation capacity, provides a useful model to assess whether candidate mechanisms operate across distinct dendritic-cell contexts [16].

Pannexin-1 (Panx1) is a large-pore channel-forming protein permeable to solutes of up to approximately 900 Da and is activated by a variety of physiological stimuli, including osmotic stress, increases in intracellular Ca^2+^, caspase-mediated cleavage of its C-terminal domain, stimulation through purinergic receptors, and redox-dependent signaling [17]. Panx1 activity has been extensively linked to ATP release and purinergic signaling, frequently in association with the P2X7 receptor, which is highly expressed in dendritic cells and other myeloid populations [18]. In addition to its plasma membrane localization, Panx1 has been reported to undergo regulated internalization and trafficking through membrane recycling pathways, supporting potential roles within intracellular compartments [19,20]. In this study, we evaluated whether Panx1 contributes to intracellular antigen handling associated with the cytosolic cross-presentation pathway. Using confocal microscopy-based analysis of antigen distribution together with functional assays of CD8^+^ T-cell activation, we examined the impact of Panx1 inhibition on antigen redistribution associated with cross-presentation and on MHC-I-restricted presentation. By combining inflammatory BMDC-like cells and the cDC1-like MUTU1940 model, and by assessing the contribution of P2X7 signaling, we aimed to define the functional relevance of Panx1 within cross-presentation under controlled experimental conditions.

## 2. Results

### 2.1. Validation of Panx1 Expression in BMDCs and MUTU1940 Cells

**Figure 1:**
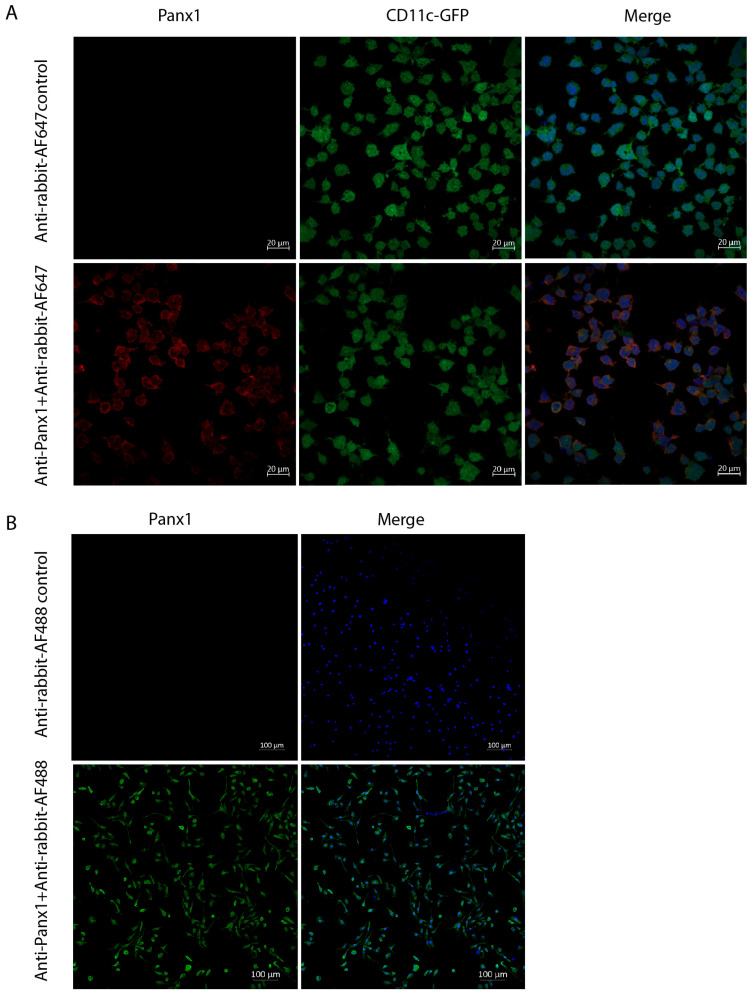
Validation of Pannexin-1 (Panx1) expression in bone marrow-derived dendritic cells (BMDCs) and MUTU1940 cells. (**A**) BMDCs were stained with an anti-Panx1 antibody followed by an Alexa Fluor 488-conjugated secondary antibody. Secondary-antibody-only controls were included to assess nonspecific fluorescence. Representative images show detectable Panx1 immunoreactivity in BMDCs, whereas control samples exhibited minimal background staining. Nuclei were counterstained with DAPI. Scale bar = 100 μm. (**B**) MUTU1940 cells expressing endogenous CD11c–green fluorescent protein (CD11c-GFP)were stained with an anti-Panx1 antibody followed by an Alexa Fluor 647-conjugated secondary antibody. Secondary-antibody-only controls were included to assess nonspecific fluorescence. Representative images show detectable Panx1 immunoreactivity in CD11c-GFP-positive MUTU1940 cells, whereas control samples exhibited negligible fluorescence signal. Scale bar = 20 μm. Representative images from 3 independent experiments; acquisition was performed using ZEN software (version 3.1.3, Zeiss).

Before evaluating the potential contribution of Panx1 to antigen processing and cross-presentation, we first sought to confirm Panx1 expression in the cellular models used throughout this study. Immunofluorescence staining was therefore performed in both BMDCs and MUTU1940 cells using an anti-Panx1 antibody together with corresponding secondary-antibody-only controls. In CD11c–green fluorescent protein (CD11c-GFP)-positive MUTU1940 cells, anti-Panx1 staining produced a readily detectable fluorescence signal Alexa Fluor 647 (AF647), whereas secondary-antibody controls showed negligible background staining (Figure 1A). Similarly, Panx1 immunoreactivity was detected in BMDCs Alexa Fluor 488 (AF488), while secondary-antibody-only controls remained essentially negative (Figure 1B). Together, these findings confirm the presence of Panx1 in both cellular models used for the subsequent functional analyses. Throughout this manuscript, the term “BMDCs” refers to dendritic-like cells generated from bone marrow cultures supplemented with GM-CSF and interleukin-4 (IL-4). These cultures predominantly consist of inflammatory monocyte-derived cells with dendritic features, rather than ontogenically defined FMS-like tyrosine kinase 3 ligand (FLT3L)-dependent cDC1 or cDC2 subsets [14].

### 2.2. Evaluation of the Effect of Pannexin-1 Inhibition on Intracellular Antigen Redistribution in Dendritic Cells

**Figure 2:**
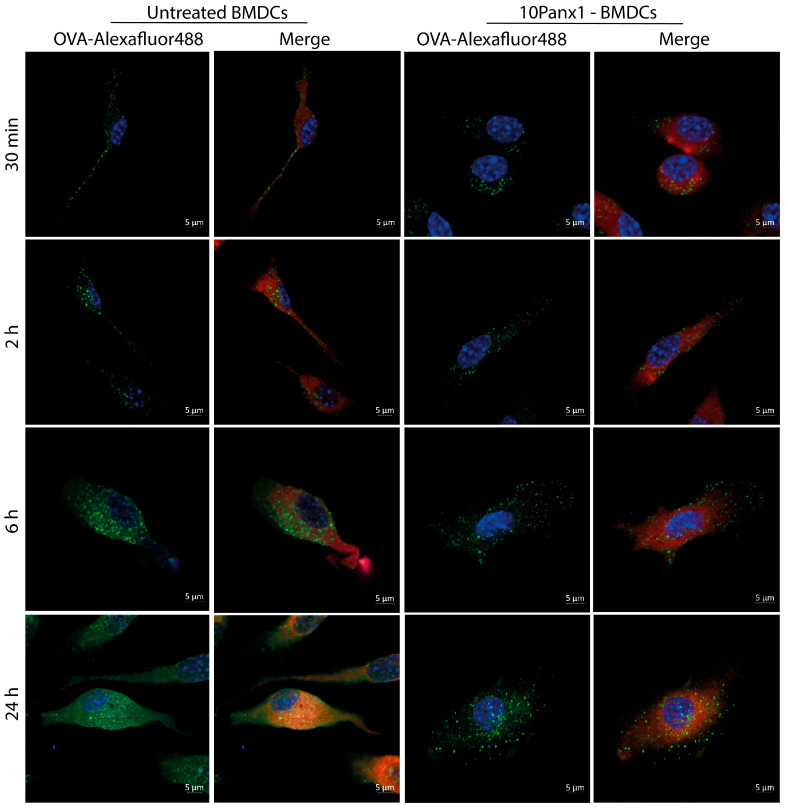
Intracellular distribution of OVA–Alexa Fluor 488 in dendritic cells treated or not with the Pannexin-1 (Panx1) inhibitor. bone marrow-derived dendritic cells (BMDCs) were incubated with ovalbumin (OVA)–Alexa Fluor 488 for 30 min, 2 h, 6 h, or 24 h in the absence or presence of the Panx1 inhibitory peptide (10Panx1). After incubation, cells were stained with CellTracker™ Red CMTPX (red) to define intracellular cellular regions and with DAPI (blue) to visualize nuclei. The green signal corresponds to OVA–Alexa Fluor 488, and the merged panels show the superposition of the three fluorescence channels. Samples were analyzed using a Carl Zeiss LSM 800 confocal microscope (Carl Zeiss Microscopy GmbH, Jena, Germany) using identical acquisition settings for all conditions. Representative images from four independent experiments are shown. Scale bar = 10 µm.

**Figure 3:**
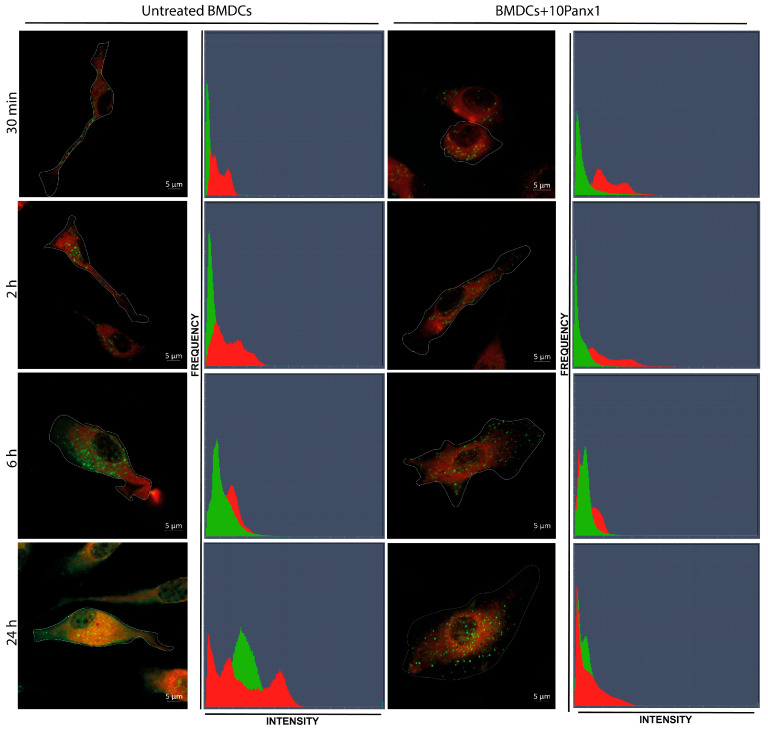
Representative fluorescence intensity histograms of ovalbumin (OVA)–Alexa Fluor 488 in bone marrow-derived dendritic cells (BMDCs). Representative confocal microscopy images and corresponding fluorescence intensity histograms showing OVA–Alexa Fluor 488 fluorescence patterns in BMDCs at 30 min, 2 h, 6 h, and 24 h of incubation. Left panels show untreated BMDCs, whereas right panels show BMDCs treated with the Panx1 inhibitory peptide (10Panx1, 200 μM). For each condition, regions of interest (ROIs) were defined on the left subpanels based on CellTracker™ Red CMTPX staining (red), which was used to define intracellular regions for fluorescence quantification. OVA–Alexa Fluor 488 fluorescence is shown in green. Fluorescence intensity histograms represent the pixel intensity distribution of OVA–Alexa Fluor 488 within CellTracker-defined ROIs. Images and histograms are representative of six independent experiments (*n* = 6). Image acquisition and histogram analysis were performed using ZEN software (version 3.1.3, Zeiss).

**Figure 4:**
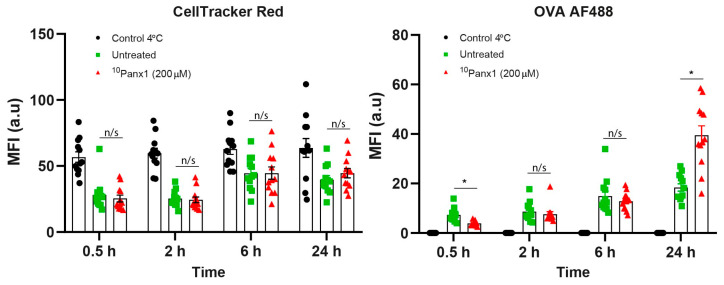
Quantification of intracellular fluorescence intensity of CellTracker Red and ovalbumin (OVA)–Alexa Fluor 488 in bone marrow-derived dendritic cells (BMDCs). BMDCs were incubated with OVA–Alexa Fluor 488 for the indicated time points (0.5, 2, 6, and 24 h) in the absence (untreated, green squares) or presence of the Panx1 inhibitory peptide 10Panx1 (200 µM, red triangles). Cells incubated at 4 °C were included as a negative control (black circles). Confocal images were analyzed to quantify mean fluorescence intensity (MFI, arbitrary units) within regions of interest (ROIs) corresponding to individual cells. Left panel: Quantification of CellTracker™ Red CMTPX fluorescence intensity, used to define intracellular ROIs and assess ROI consistency across conditions and time points. Right panel: Quantification of OVA–Alexa Fluor 488 fluorescence intensity measured within CellTracker Red-defined ROIs. ROIs were manually delineated based exclusively on the CellTracker Red signal, background subtraction was applied uniformly, and identical acquisition and analysis parameters were maintained for all conditions. Each data point represents an individual cell. Quantification was performed on randomly selected cells from six independent experiments (*n* = 6). Data are presented as mean ± SEM. Statistical comparisons were restricted to predefined biologically relevant groups and analyzed using the Mann–Whitney test. Statistical significance is indicated as shown (* *p* < 0.05; n/s, not significant).

The first objective of this study was to assess whether Panx1 contributes to the intracellular handling of exogenous antigens during cross-presentation, a process functionally associated with the cytosolic pathway [21]. This analysis was based on the hypothesis that Panx1, a large-conductance channel involved in molecular exchange across cellular membranes, could influence intracellular antigen redistribution following phagocytic uptake, thereby modulating downstream antigen-processing events associated with MHC class I presentation. In support of this rationale, previous studies have reported a role for Panx1 in facilitating the cytosolic access of muramyl dipeptide (MDP) after phagocytosis in macrophages [22]. However, whether a similar mechanism operates during antigen cross-presentation in dendritic cells has not been explored. Therefore, the experiments described below were designed to evaluate intracellular antigen redistribution associated with cross-presentation rather than to directly demonstrate cytosolic escape. To address this question, the temporal intracellular distribution of ovalbumin (OVA)–Alexa Fluor 488 was analyzed in untreated BMDCs and in cells treated with the Panx1 inhibitory peptide using confocal microscopy. At 30 min of incubation, both conditions displayed a predominantly punctate antigen signal confined to vesicular structures, consistent with an early phase of endocytic uptake. This vesicular pattern was maintained at 2 h, with no evident differences between untreated and 10Panx1-treated cells, suggesting that Panx1 inhibition does not affect the initial internalization of the antigen. From 6 h onward, untreated BMDCs exhibited a progressive change in antigen distribution, characterized by a more dispersed and homogeneous intracellular signal that became more pronounced at 24 h (Figure 2, left panel). This qualitative shift was reflected in the fluorescence intensity histograms (Figure 3), which showed a broadening of the OVA–Alexa Fluor 488 intensity distribution within regions defined by CellTracker Red, suggesting redistribution of the antigen beyond discrete vesicular compartments. Fluorescence intensity histograms were generated using thresholds defined from phagocytosis control conditions (Supplementary Figure S1). Quantitative analysis of the mean fluorescence intensity (MFI) of OVA within CellTracker Red-defined regions revealed a time-dependent increase in both conditions; however, 10Panx1-treated cells displayed a significantly higher OVA MFI at 24 h compared with untreated cells (Figure 4). In contrast, CellTracker Red fluorescence remained stable across experimental conditions and time points, indicating that the intracellular regions used for fluorescence quantification were comparable between untreated and 10Panx1-treated cells. Because fluorescence quantification was performed exclusively within regions of interest (ROIs) defined by CellTracker Red signal, the increased OVA–Alexa Fluor 488 signal observed following Panx1 inhibition is unlikely to reflect differences in ROI definition or cell-associated fluorescence and is instead consistent with intracellular retention or accumulation of antigen during late stages of antigen processing. Nevertheless, because these analyses rely on fluorescence-based approaches, the results are interpreted as consistent with altered intracellular antigen redistribution and retention during late stages of antigen processing, rather than as definitive evidence of cytosolic escape.

**Figure 5:**
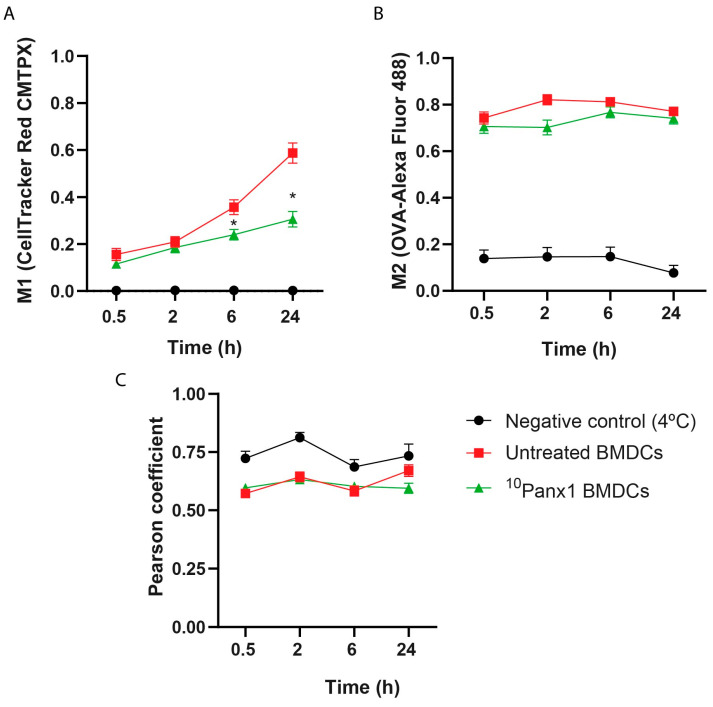
Quantitative colocalization analysis between ovalbumin (OVA)–Alexa Fluor 488 and CellTracker Red CMTPX in bone marrow-derived dendritic cells (BMDCs). Untreated BMDCs (red), BMDCs treated with the Pannexin-1 (Panx1) inhibitory peptide (10Panx1, 200 µM; green), and negative-control cells maintained at 4 °C (black) were incubated with OVA–Alexa Fluor 488 for the indicated time points (0.5, 2, 6, and 24 h) and analyzed by confocal microscopy. (**A**) Manders’ M1 coefficient. (**B**) Manders’ M2 coefficient, representing the fraction of OVA–Alexa Fluor 488 fluorescence overlapping with CellTracker Red signal. (**C**) Pearson’s correlation coefficient, reflecting the overall fluorescence intensity correlation between OVA–Alexa Fluor 488 and CellTracker Red signals. Quantitative analysis was performed on regions of interest (ROIs) corresponding to individual cells and defined exclusively based on CellTracker Red signal. Background subtraction and fluorescence thresholding were applied uniformly within each experiment. Multiple fields were analyzed per condition from six independent experiments (*n* = 6). Data are presented as mean ± SEM. Images were acquired and analyzed using ZEN software version 3.1.3 (Zeiss). Statistical comparisons were restricted to predefined biologically relevant groups as described in the Section 4. Asterisks (*) indicate statistically significant differences (* *p* < 0.05).

Quantitative colocalization analysis between OVA–Alexa Fluor 488 and CellTracker Red CMTPX was performed on confocal images acquired under identical acquisition parameters for all experimental conditions. Regions of interest (ROIs) corresponding to individual cells were defined based exclusively on CellTracker Red signal, excluding extracellular fluorescence. Background subtraction and fluorescence thresholding were applied uniformly within each experiment, and multiple cells were analyzed per condition across independent experiments (*n* = 6). The Manders M1 coefficient increased progressively over time in untreated BMDCs, reaching its highest values at 24 h (Figure 5A). In contrast, BMDCs treated with the Panx1 inhibitory peptide displayed significantly lower M1 values at 6 and 24 h (*p* < 0.05), indicating reduced spatial overlap between OVA fluorescence and CellTracker-defined intracellular regions at later time points. As expected, the negative control incubated at 4 °C showed M1 values close to zero, consistent with minimal active antigen internalization. The Manders M2 coefficient, reflecting the proportion of OVA fluorescence overlapping with CellTracker Red signal, remained high and relatively stable across time points and experimental conditions (Figure 5B). This finding indicates that, once internalized, most of the antigen signal remained associated with intracellular regions defined by CellTracker Red, regardless of Panx1 inhibition. Analysis of the Pearson correlation coefficient revealed no significant differences between untreated and inhibitor-treated cells at any time point (Figure 5C), indicating comparable overall fluorescence intensity correlations between OVA and CellTracker Red signals across conditions. Taken together, these results support altered intracellular antigen redistribution following Panx1 inhibition. However, because CellTracker Red was used exclusively to define intracellular cellular regions and not to directly identify a cytosolic compartment, these colocalization analyses should not be interpreted as definitive evidence of cytosolic escape or phagosome-to-cytosol transfer.

### 2.3. Evaluation of the Role of Panx1 in OVA Cross-Presentation

**Figure 6:**
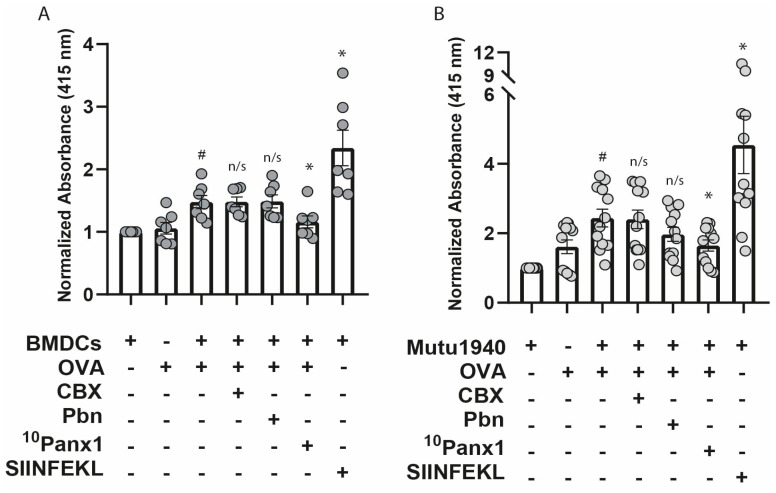
Inhibition of Pannexin-1 (Panx1) decreases cross-presentation of ovoalbumin (OVA) antigens in dendritic cells. (**A**) Bone marrow-derived dendritic cells (BMDCs) and (**B**) MUTU1940 cells were pre-incubated with the Panx1 inhibitors 10Panx1, carbenoxolone (CBX), or probenecid (PBN) for 30 min, followed by incubation with full-length OVA for 24 h. Cells were then extensively washed and co-cultured with B3Z hybridoma cells for 48 h. B3Z activation was quantified by measuring β-galactosidase activity using ONPG as substrate, and absorbance was recorded at 415 nm. Data were normalized to the condition “BMDCs (or MUTU1940) + B3Z without OVA” and are presented as mean ± SEM from six independent experiments (*n* = 6). SIINFEKL was used as positive control and was added directly to dendritic cells prior to co-culture. Statistical comparisons were restricted to predefined biologically relevant groups. B3Z activation induced by OVA was compared with the corresponding inhibitor-treated conditions, and OVA-treated cells were compared with the no-OVA negative control. The symbols “+” and “−” indicate the presence or absence, respectively, of the indicated antigen, peptide, inhibitor, or treatment condition. Differences were evaluated using a one-tailed non-parametric Mann–Whitney test. * *p* < 0.05 versus OVA-treated cells; # *p* < 0.05 versus the condition without OVA; n/s, not significant.

After evaluating the effects of Panx1 inhibition on intracellular antigen redistribution, we next examined whether pharmacological inhibition of Panx1 affected the functional outcome of antigen cross-presentation. Because antigen cross-presentation capacity can vary across dendritic cell models [23], these experiments were performed both in primary GM-CSF/IL-4-derived BMDCs and in the MUTU1940 cell line, a well-established cDC1-like model, to assess whether the effects of Panx1 inhibition on cross-presentation are conserved across distinct dendritic cell models. To this end, antigen-presenting cells were pretreated with different Panx1-targeting inhibitors, including the blocking peptide 10Panx1, carbenoxolone (CBX), or probenecid (PBN), and subsequently pulsed with full-length OVA for 24 h. Cells were then co-cultured with SIINFEKL-specific B3Z reporter cells for 48 h, and B3Z activation was quantified by measuring β-galactosidase activity using ONPG as substrate (absorbance at 415 nm), following established cross-presentation assay formats [24]. In BMDCs, incubation with OVA induced robust B3Z activation compared with the baseline condition in which dendritic cells were co-cultured with B3Z in the absence of antigen (Figure 6A). Treatment with the Panx1 inhibitory peptide 10Panx1 resulted in a significant reduction in B3Z activation (*p* < 0.05) relative to OVA-treated controls, consistent with the alterations in intracellular antigen handling observed in confocal microscopy analyses. In contrast, treatment with CBX or PBN did not significantly alter B3Z activation under these conditions. As expected, direct loading with the SIINFEKL peptide induced maximal B3Z activation, confirming the responsiveness of the reporter system, whereas negative control conditions did not elicit detectable β-galactosidase activity.

In MUTU1940 cells, OVA pulsing similarly induced strong B3Z activation (Figure 6B). In this model, treatment with 10Panx1 significantly reduced B3Z activation compared with untreated OVA-pulsed cells (*p* < 0.05). In addition, probenecid treatment also resulted in a significant decrease in B3Z activation, whereas carbenoxolone had no detectable effect. As in BMDCs, SIINFEKL loading produced robust B3Z activation, and control conditions lacking antigen did not induce reporter activity. These results show that pharmacological inhibition of Panx1 is associated with reduced functional cross-presentation of soluble OVA in both primary BMDCs and MUTU1940 cells. While the overall pattern of Panx1 dependence was conserved across both models, differences in the effects of probenecid were observed, suggesting possible model-dependent differences in inhibitor sensitivity.

### 2.4. Evaluation of the Role of the P2X7 Receptor in OVA Cross-Presentation

After establishing that pharmacological inhibition of Panx1 is associated with reduced OVA cross-presentation in both primary BMDCs and MUTU1940 cells, we next examined whether this effect was functionally linked to signaling through the purinergic receptor P2X7. P2X7 has been described as an upstream regulator of Panx1 activity in several cellular contexts, particularly in processes involving ATP release, inflammasome activation, and inflammatory signaling [17,18,25]. To assess the potential contribution of P2X7 to antigen cross-presentation, we employed complementary pharmacological and genetic approaches. Specifically, we evaluated the impact of P2X7 blockade using selective inhibitors and assessed OVA cross-presentation in dendritic cells derived from P2X7-deficient (P2X7 KO) mice, using wild-type (WT) cells as controls. These analyses were performed in GM-CSF/IL-4-derived BMDC cultures and, in parallel, in the MUTU1940 cell line, a well-established cDC1-like model, allowing comparison across distinct dendritic cell systems.

### 2.5. Pharmacological Inhibition of P2X7 in BMDCs and MUTU1940

**Figure 7:**
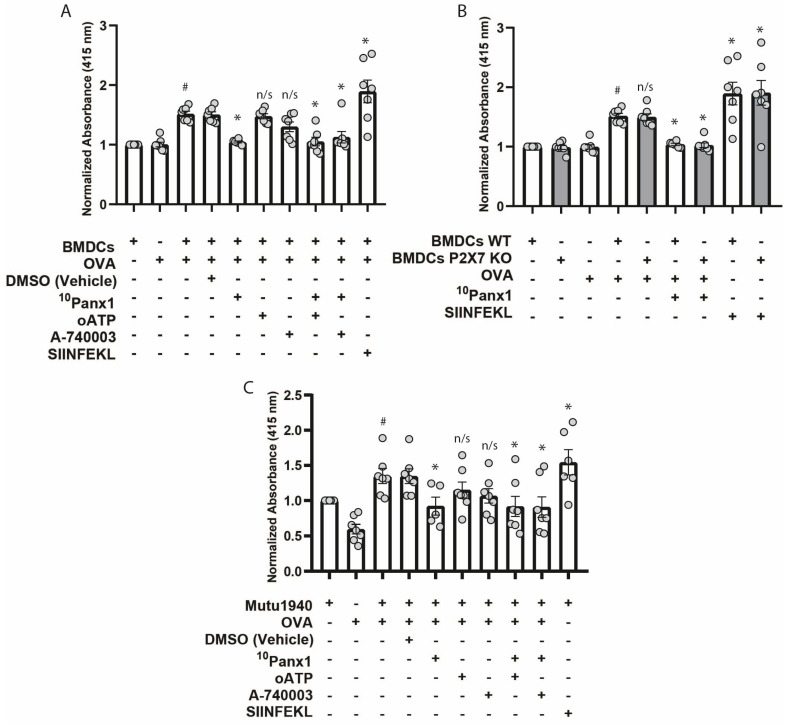
Pannexin-1 (Panx1) inhibition reduces antigen cross-presentation independently of P2X7 activity in bone marrow-derived dendritic cells (BMDCs) and MUTU1940 cells. (**A**) Bone marrow-derived dendritic cells (BMDCs) were pulsed with soluble ovoalbumin (OVA) and treated with vehicle (DMSO), the Panx1 inhibitory peptide 10Panx1 (200 µM), the P2X7 inhibitors oATP (200 µM) or A-740003 (100 µM), or the indicated combinations. Following antigen processing, BMDCs were co-cultured with B3Z hybridoma cells, and β-galactosidase activity was quantified by measuring absorbance at 415 nm. Data are shown as normalized absorbance relative to control conditions. (**B**) BMDCs derived from wild-type (WT) or P2X7-deficient (P2X7 KO) mice were pulsed with OVA or SIINFEKL and co-cultured with B3Z cells in the presence or absence of 10Panx1. Comparable B3Z activation was observed between WT and P2X7 KO BMDCs under basal conditions, whereas Panx1 inhibition reduced B3Z activation in both genotypes. (**C**) MUTU1940 cells were treated with OVA, vehicle, 10Panx1, oATP, A-740003, or the indicated combinations and subsequently co-cultured with B3Z cells. β-galactosidase activity was quantified as described in panel A. Bars represent mean ± SEM, and individual points correspond to independent biological replicates. Statistical comparisons were restricted to predefined biologically relevant groups. B3Z activation induced by OVA was compared with the corresponding inhibitor-treated conditions, and antigen-containing conditions were compared with unstimulated controls. The symbols “+” and “−” indicate the presence or absence, respectively, of the indicated antigen, peptide, inhibitor, or treatment condition. Statistical analysis was performed using a one-tailed non-parametric Mann–Whitney test, as described in the Section 4. * *p* < 0.05; n/s, not significant; # *p* < 0.05 versus unstimulated control.

In BMDCs (Figure 7A), inhibition of Panx1 with 10Panx1 significantly reduced B3Z activation (*p* < 0.05), indicating a functional contribution of Panx1 to antigen cross-presentation. In contrast, inhibition of P2X7 using oATP or A-740003 did not result in a comparable reduction in β-galactosidase activity. Moreover, combined inhibition of Panx1 and P2X7 did not produce an additive effect relative to Panx1 inhibition alone. These results suggest that, under the experimental conditions tested, Panx1-dependent cross-presentation does not require concurrent P2X7 activity. Similar results were obtained in MUTU1940 cells (Figure 7C). In this model, treatment with 10Panx1 significantly reduced B3Z activation, whereas inhibition of P2X7 with oATP or A-740003 led to modest and non-significant changes compared with untreated controls. As observed in BMDCs, combined inhibition of Panx1 and P2X7 did not further reduce cross-presentation, supporting the notion that Panx1 function in this context is largely independent of P2X7 signaling. To further evaluate the contribution of P2X7, BMDCs derived from WT and P2X7-deficient mice were compared (Figure 7B). In the absence of inhibitors, WT and P2X7-KO BMDCs exhibited comparable levels of B3Z activation, indicating that P2X7 deficiency does not impair basal cross-presentation capacity. Inhibition of Panx1 with 10Panx1 resulted in a similar reduction in B3Z activation in both genotypes, further supporting that Panx1-dependent cross-presentation is largely preserved in the absence of P2X7 under these experimental conditions. While pharmacological inhibition of P2X7 produced mild effects in some settings, the absence of this receptor did not significantly compromise antigen-specific CD8^+^ T-cell activation.

To determine whether the effects of Panx1 inhibition on cross-presentation could be attributed to nonspecific alterations in dendritic cell viability or activation status, BMDCs and MUTU1940 cells were treated with 10Panx1 under the same experimental conditions used throughout this study. Assessment of cell viability revealed no significant differences between untreated and 10Panx1-treated cells in either model (Supplementary Figures S3A and S4A). Likewise, treatment with 10Panx1 did not significantly affect the expression of the activation marker CD40 under basal conditions or following LPS stimulation in BMDCs and MUTU1940 cells (Supplementary Figures S3 and S4; B,C). Taken together, these results indicate that 10Panx1 at the concentration used in this study does not compromise dendritic cell viability or impair LPS-induced activation. Therefore, the reduction in OVA cross-presentation observed following Panx1 inhibition is unlikely to result from cytotoxic effects or a generalized defect in dendritic cell activation, supporting a more specific role for Panx1 in antigen-processing events associated with cross-presentation.

## 3. Discussion

Cross-presentation of exogenous antigens through MHC class I molecules requires a series of intracellular processing events that enable efficient activation of cytotoxic CD8^+^ T lymphocytes. Among the pathways described to support this process, the cytosolic route has been extensively studied because it involves proteasomal degradation of antigenic material prior to peptide loading onto MHC-I molecules [3,4]. Consequently, the intracellular mechanisms that regulate antigen handling and processing have emerged as critical determinants of effective antiviral and antitumor immune responses. Consistent with this concept, several recent reviews have highlighted intracellular antigen trafficking and processing as key regulatory steps controlling cross-presentation efficiency [26].

For more than a decade, components of the endoplasmic reticulum-associated degradation (ERAD) machinery, most notably the Sec61 translocon, were proposed as major contributors to antigen processing during cross-presentation. Early studies reported enrichment of ERAD-associated proteins, including Sec61 and the E3 ligase Hrd1, in antigen-containing phagosomes, supporting the concept of ER–phagosome cooperation during antigen processing [5,27]. Furthermore, functional studies such as those by Zehner et al. [7] demonstrated that interference with Sec61 activity reduced cross-presentation efficiency. However, subsequent evidence challenged the view that Sec61 represents the sole mechanism responsible for antigen transfer to cytosol-associated processing pathways. In particular, pharmacological inhibition of Sec61 with mycolactone impaired cross-presentation without preventing antigen redistribution to downstream processing compartments, suggesting that additional mechanisms contribute to this process [6]. Collectively, these observations indicate that although ERAD-related components participate in cross-presentation, additional molecular pathways are likely involved in regulating intracellular antigen handling and processing events associated with efficient MHC-I presentation [28].

In parallel with channel-based transport models, mechanisms involving controlled vesicular permeabilization have also been proposed to facilitate antigen processing during cross-presentation. Among these, Perforin-2 (MPEG-1) has been described as a pore-forming effector capable of promoting antigen escape from endosomal compartments [10]. While this mechanism provides an alternative route for antigen processing, it is associated with physiological constraints, as disruption of vesicular integrity may trigger local inflammatory responses and compromise cellular homeostasis, potentially limiting its relevance in steady-state or vaccination settings. In this context, the present study examined the contribution of Panx1 to intracellular antigen handling during cross-presentation and subsequent CD8^+^ T-cell activation. Panx1 is a large-pore channel permeable to solutes of up to approximately 900 Da and can be activated by a variety of physiological stimuli, including osmotic stress, elevations in intracellular Ca^2+^, caspase-mediated cleavage of its C-terminal domain, stimulation through purinergic receptors such as P2X7, and NOX2-dependent oxidative signals [17,29,30]. Although the precise subcellular localization of Panx1 under antigen uptake conditions was not directly assessed, previous studies have demonstrated that Panx1 can undergo regulated internalization and trafficking from the plasma membrane to intracellular compartments, including endosomal and recycling pathways [19,20,31]. Using confocal microscopy combined with functional B3Z reporter assays, we found that pharmacological inhibition of Panx1 was associated with altered intracellular redistribution of antigen and a concomitant reduction in SIINFEKL-dependent β-galactosidase activation. Importantly, these effects are interpreted as reflecting altered intracellular antigen processing associated with cross-presentation rather than direct translocation of intact OVA through the Panx1 channel, which would be incompatible with the known biophysical properties of Panx1. These effects were consistently observed in both BMDCs and MUTU1940 cells, despite the higher intrinsic cross-presentation capacity of the cDC1-like MUTU1940 model. Furthermore, inhibition of Panx1 resulted in a comparable reduction in antigen-specific CD8^+^ T-cell activation across both systems, indicating that its contribution is preserved in distinct dendritic-cell contexts. In parallel, neither pharmacological inhibition nor genetic ablation of P2X7 substantially modified the effect of Panx1 inhibition on cross-presentation, suggesting that Panx1 function in this setting operates largely independently of classical P2X7-mediated purinergic signaling. This observation is notable given that the Panx1–P2X7 axis has been predominantly studied in the context of inflammatory signaling, ATP release, and cell death pathways rather than antigen processing and presentation. While these findings do not exclude context-dependent interactions between Panx1 and purinergic receptors, they indicate that Panx1-mediated modulation of cross-presentation does not require P2X7 activity under the experimental conditions examined. Taken together, our data support a model in which Panx1 contributes to intracellular antigen-processing events associated with cross-presentation without necessitating overt vesicular disruption or loss of cellular integrity. In this regard, Panx1 may represent a component of a controlled, non-lytic mechanism that facilitates antigen handling during cross-presentation. These observations are consistent with previous conceptual frameworks proposing the existence of alternative routes contributing to antigen processing during cross-presentation [32,33], and extend prior work by identifying Panx1 as a functionally relevant contributor to CD8^+^ T-cell activation. Moreover, the absence of significant effects on dendritic-cell viability or LPS-induced activation following 10Panx1 treatment supports the interpretation that the reduction in cross-presentation is unlikely to result from nonspecific cytotoxicity or generalized impairment of dendritic-cell function. Overall, our findings reinforce the idea that regulated, non-destructive pathways can support efficient antigen processing and presentation while preserving dendritic-cell viability and activation status. A limitation of the present study is that cytosolic access of antigenic material was not directly demonstrated using an orthogonal assay such as selective permeabilization, cytosolic fractionation, or reporter-based escape approaches. Therefore, the fluorescence redistribution patterns observed here should not be interpreted as definitive evidence of phagosome-to-cytosol transfer or cytosolic escape. Rather, our findings support a role for Panx1 in intracellular antigen-handling and -processing events functionally associated with cross-presentation. Future studies incorporating direct cytosolic-access assays will be required to define the precise mechanistic contribution of Panx1.

Our findings may also help explain previous observations indicating that inhibition of Sec61 reduces cross-presentation without completely preventing antigen access to downstream processing pathways [6]. Such studies have suggested that additional mechanisms contribute to intracellular antigen processing beyond the classical ERAD machinery. In this context, Panx1 may represent one component of a broader network of pathways that collectively regulate antigen processing during cross-presentation. Although the precise mechanisms governing Panx1 activity within antigen-processing compartments remain unknown, previous studies have shown that Panx1 can be regulated through multiple stimuli [34]. Therefore, it is conceivable that Panx1 responds to local signals associated with endosomal maturation, including changes in membrane composition, redox status, or luminal conditions. Future studies will be required to determine whether such mechanisms contribute to Panx1-dependent regulation of antigen processing in dendritic cells.

An additional question arising from our findings concerns the nature of the antigen-processing events that may precede Panx1-dependent regulation of cross-presentation. Previous studies have shown that enzymes such as γ-interferon-inducible lysosomal thiol reductase (GILT) contribute to antigen processing by promoting the reduction in disulfide bonds within endocytic compartments, thereby facilitating the generation of antigenic intermediates suitable for downstream processing [35]. Although the present study did not evaluate the relationship between GILT and Panx1, it is conceivable that redox-dependent antigen processing influences intracellular antigen redistribution events associated with Panx1 function. Likewise, the mechanisms responsible for Panx1 localization and activation within antigen-processing compartments remain unknown. Previous studies have demonstrated intracellular trafficking of Panx1 through endosomal and recycling pathways [36], providing a potential framework for future investigations aimed at defining how Panx1 participates in antigen processing during cross-presentation.

The comparable reduction in cross-presentation observed following Panx1 inhibition in both BMDCs and MUTU1940 cells suggests that Panx1 contributes to a shared aspect of dendritic-cell biology involved in antigen processing. Consistent with this interpretation, Panx1 is broadly expressed across immune cell types and has been implicated in diverse functions, including ATP release, inflammasome activation, and danger-signal propagation [22,37,38,39]. Together, these observations support the view that Panx1 represents a conserved molecular component that may participate in intracellular antigen-processing events relevant to cross-presentation, although the precise molecular mechanisms remain to be determined.

The higher magnitude of B3Z activation observed in MUTU1940 cells compared with BMDCs is consistent with the superior cross-presentation capacity previously described for cDC1-like cells [40]. Nevertheless, the inhibitory effect of Panx1 blockade was consistently observed in both models, indicating that the contribution of Panx1 is preserved across distinct dendritic-cell contexts despite differences in their intrinsic cross-presentation efficiency.

**Figure 8:**
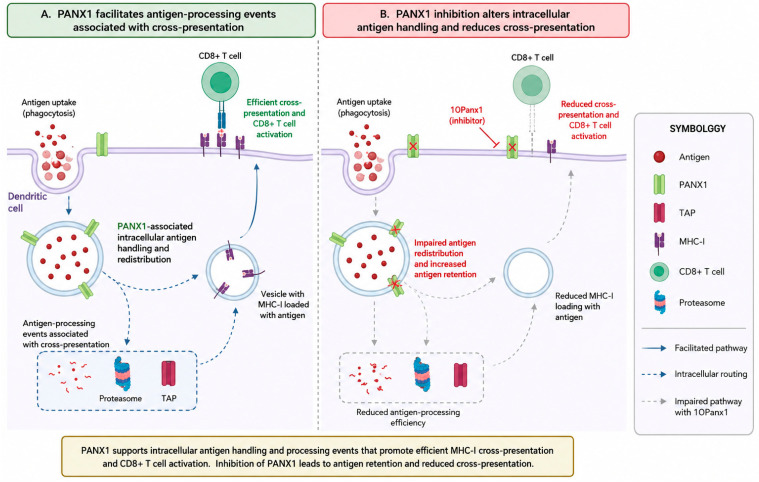
Proposed model of Pannexin-1 (Panx1) involvement in antigen-processing events associated with cross-presentation. Following antigen uptake, Panx1 is proposed to modulate intracellular antigen handling and redistribution during antigen processing, thereby supporting efficient major histocompatibility complex class I (MHC-I)-restricted cross-presentation and CD8^+^ T-cell activation. Pharmacological inhibition of Panx1 promotes intracellular antigen retention and reduces antigen-specific CD8^+^ T-cell activation. This model summarizes the experimental observations and should not be interpreted as direct evidence of phagosome-to-cytosol transfer or cytosolic escape. Figure prepared by the authors.

Taken together, the findings of this study provide functional evidence that pharmacological inhibition of Panx1 modulates the efficiency of antigen cross-presentation. Our results identify Panx1 as a functionally relevant element influencing intracellular antigen redistribution and subsequent MHC-I-restricted CD8^+^ T-cell activation. The consistent reduction in B3Z activation observed following Panx1 inhibition in both primary BMDCs and the MUTU1940 dendritic cell line indicates that this effect is preserved across distinct dendritic-cell models under the experimental conditions examined. While the present study does not establish direct genetic causality, the combined imaging and functional data support a model in which Panx1 activity contributes to antigen-processing events associated with cross-presentation without overt disruption of vesicular integrity. In this context, our findings expand current conceptual frameworks of intracellular antigen processing by highlighting Panx1 as a potential modulatory component acting alongside previously proposed mechanisms involved in cross-presentation. Based on these observations, we propose a schematic model summarizing a potential role for Panx1 in intracellular antigen-processing events associated with cross-presentation (Figure 8).

Although pharmacological inhibition represents a useful approach to interrogating Panx1 function, the present study remains limited by the absence of complementary genetic loss-of-function strategies. While 10Panx1 is widely used and validated as a Panx1 inhibitor, pharmacological approaches cannot completely exclude off-target effects. In addition, although Panx1 expression was confirmed in both BMDCs and MUTU1940 cells, its precise subcellular localization during antigen processing was not directly assessed. Another important limitation is that intracellular antigen redistribution was evaluated using fluorescence-based approaches, which do not directly demonstrate phagosome-to-cytosol transfer or cytosolic access of antigenic material. Therefore, the observed fluorescence patterns should be interpreted as evidence of altered intracellular antigen handling rather than definitive proof of cytosolic escape. Future studies incorporating genetic models, direct cytosolic-access assays, and in vivo approaches will be required to define the precise mechanistic role of Panx1 during cross-presentation. Future studies should also determine whether the mechanisms described here extend to cell-associated antigens and more physiologically relevant models of cross-presentation.

## 4. Materials and Methods

### 4.1. Animals

Healthy male and female mice from the C57BL/6 strain or the B6.129P2-P2RX7^tm1Gab^ (P2X7 KO) line, aged 6–12 weeks, were obtained from the “Eduardo Morales Santos” Research Center at the University of Santiago de Chile. Animals were housed under controlled temperature conditions and maintained with ad libitum access to food and water, under a 12 h light/12 h dark cycle. All procedures involving animals were reviewed and approved by the Institutional Animal Care and Use Committee of the University of Santiago de Chile (approval number 510.2024). Experimental procedures were conducted in accordance with international guidelines for the recognition of pain, distress, and discomfort in laboratory animals.

### 4.2. Cell Lines

The Mutu1940 dendritic cell line, representative of the Conventional type 1 dendritic cells (cDC1) subset, was cultured in RPMI-1640 medium (Thermo Fisher Scientific, Waltham, MA, USA). Mutu DCs [41], kindly provided by Dr. Hans Acha-Orbea (University of Lausanne), stably express the enhanced green fluorescent protein (eGFP) gene under the control of the CD11c promoter. The culture medium for HEK293 cells was supplemented with 15% fetal bovine serum (FBS; Gibco, Thermo Fisher Scientific, Waltham, MA, USA). All cell culture media were supplemented with 100 U/mL penicillin, 100 μg/mL streptomycin, and 2.5 μg/mL amphotericin B (Sigma-Aldrich, St. Louis, MO, USA). Cell lines were maintained at 37 °C in a humidified atmosphere containing 5% CO_2_.

### 4.3. Primary Culture of Bone Marrow-Derived Dendritic Cells (BMDCs)

Bone marrow-derived dendritic cells (BMDCs) were generated from mouse bone marrow cultures supplemented with granulocyte–macrophage colony-stimulating factor (GM-CSF; BioLegend, San Diego, CA, USA)and interleukin-4 (IL-4; BioLegend, San Diego, CA, USA). Under these conditions, the resulting cultures predominantly consist of inflammatory BMDC-like cells with dendritic features, rather than bona fide FMS-like tyrosine kinase 3 ligand (FLT3L)-dependent cDC1 or cDC2 subsets. Throughout this manuscript, these cells are referred to as “BMDCs” unless otherwise specified. Primary cultures of BMDCs were generated from bone marrow precursors obtained from wild-type (WT) C57BL/6 mice and P2X7-deficient (P2X7KO) mice of both sexes, as previously described, with minor modifications [24]. Mice were euthanized by cervical dislocation, and femurs and tibias were collected under sterile conditions. The epiphyses were removed, and the bones were centrifuged at 10,000× *g* for 40 s to flush the bone marrow into RPMI medium. The pellet was resuspended in ACK erythrocyte lysis buffer (0.15 M NH_4_Cl, 10 mM KHCO_3_, 0.1 mM EDTA; Sigma-Aldrich, St. Louis, MO, USA) for 5 min with gentle agitation at room temperature, followed by centrifugation at 300× *g* for 7 min. The resulting cell pellet was resuspended and seeded in 100 mm culture dishes (1.0 × 10^6^ cells per mL of complete medium) in RPMI-1640 supplemented with 10% FBS, penicillin (100 U/mL)/streptomycin (100 μg/mL), 1 mM sodium pyruvate, 1 mM L-glutamine, 1% non-essential amino acids, and 10 ng/mL GM-CSF and IL-4). After two days, 75% of the culture medium was replaced. On days 4 and 6, the medium was fully replaced with fresh RPMI supplemented with out GM-CSF and IL-4. Cells were harvested and used for experimental assays on day 7 of culture.

### 4.4. Transfer of OVA–Alexa Fluor 488 to BMDCs

BMDCs were seeded at a density of 3 × 10^5^ cells per well in 24-well plates containing sterile coverslips and incubated overnight under standard culture conditions (37 °C, 5% CO_2_). The following day, cells were stained with 5 µM CellTracker™ Red CMTPX (Thermo Fisher Scientific, Waltham, MA, USA) according to the manufacturer’s instructions. After staining, BMDCs were pre-incubated with the Panx1 inhibitory peptide 10Panx1 (200 µM; Tocris Bioscience, Bristol, UK) for 1 h under the same culture conditions. Subsequently, cells were exposed to ovoalbumin (OVA)–Alexa Fluor 488 (Thermo Fisher Scientific, Waltham, MA, USA) and incubated for 30 min, 2 h, 6 h, or 24 h to assess antigen uptake and intracellular distribution at early and late stages of the phagocytic process. At the end of each incubation period, cells were fixed with 2% paraformaldehyde (PFA; Sigma-Aldrich, St. Louis, MO, USA) for 10 min, incubated with 200 mM ammonium chloride (Sigma-Aldrich, St. Louis, MO, USA) for 10 min, and stained with DAPI (0.1 µg/mL; Thermo Fisher Scientific, Waltham, MA, USA) for 5 min to label nuclei. Three washes with filtered PBS were performed between each step. Coverslips were mounted using Fluoromount-G (Thermo Fisher Scientific, Waltham, MA, USA), and samples were examined using a Carl Zeiss LSM 800 confocal microscope (Carl Zeiss Microscopy GmbH, Jena, Germany).

### 4.5. Confocal Image Acquisition and Quantitative Image Analysis

Confocal imaging was performed using a Carl Zeiss LSM 800 confocal microscope (Carl Zeiss Microscopy GmbH, Jena, Germany) equipped with a 63× oil-immersion objective. All images were acquired under identical acquisition settings (laser power, detector gain, pinhole size, and offset) for all experimental conditions within each independent experiment. Z-stack images spanning the entire cellular volume were collected and used for subsequent analyses. Orthogonal (XZ and YZ) views were generated from the corresponding Z-stacks to verify the intracellular localization of antigen signal within the cellular volume.

Quantitative image analysis was performed using ZEN software (version 3.13, Carl Zeiss). Regions of interest (ROIs) corresponding to individual cells were manually defined based exclusively on the CellTracker™ Red CMTPX signal, to define intracellular cellular regions, thereby excluding extracellular fluorescence. Background subtraction was applied uniformly using signal levels obtained from cell-free regions, and fluorescence thresholds were kept constant within each experiment. Colocalization analyses between OVA–Alexa Fluor 488 and the cytosolic marker were performed on thresholded images to calculate Manders’ coefficients M1 and M2, as well as Pearson’s correlation coefficient. M1 represents the fraction of the cytosolic compartment containing antigen signal, whereas M2 reflects the fraction of total antigen signal associated with CellTracker-defined intracellular regions. Pearson’s coefficient was used to assess global intensity correlation between channels.

### 4.6. Fluorescence Intensity Histograms, Phagocytosis Controls, and Threshold Definition

Fluorescence intensity histograms of OVA–Alexa Fluor 488 and CellTracker™ Red signals were generated from the same cytosol-defined ROIs using ZEN software (version 3.13). Histograms were used to assess the distribution and spread of antigen-associated fluorescence within individual cells over time. Early time points (30 min and 2 h) were used as internal phagocytosis controls to establish baseline antigen uptake and vesicle-confined signal profiles, allowing for the definition of intensity thresholds applied consistently to subsequent analyses. These controls were used to discriminate between background signal, punctate vesicular antigen localization, and broader intracellular redistribution observed at later time points. Orthogonal views derived from Z-stack images were used to confirm that OVA–Alexa Fluor 488 signal detected within CellTracker-defined ROIs was located inside the cellular volume rather than at the cell surface or in extracellular compartments (Supplementary Figures S1 and S2).

### 4.7. Pharmacological Inhibition and Cross-Presentation Assays

Mutu1940 cells or BMDCs were seeded at a density of 1 × 10^5^ cells per well in U-bottom 96-well plates and incubated overnight under standard culture conditions. Cells were then pre-incubated for 1 h with the Panx1 inhibitors carbenoxolone (CBX, 10 µM; igma-Aldrich, St. Louis, MO, USA), probenecid (PBN, 350 µM; Sigma-Aldrich, St. Louis, MO, USA), or the inhibitory peptide 10Panx1 (200 µM; Tocris Bioscience), and/or with the P2X7 inhibitors A-740003 (100 µM; Tocris Bioscience, Bristol, UK) or oxidized ATP (oATP, 200 µM; Sigma-Aldrich, St. Louis, MO, USA). Following pharmacological blockade, antigen-presenting cells were pulsed with soluble OVA (250 µg/mL; InvivoGen, San Diego, CA, USA) and incubated for 18–24 h. Plates were then centrifuged at 500× *g* for 2 min, supernatants were removed, and cells were co-cultured with 2 × 10^5^ B3Z hybridoma cells for 48 h under the same culture conditions. After co-culture, plates were centrifuged again under the same conditions and incubated with developing solution (5 mM o-nitrophenyl-β-D-galactopyranoside [ONPG]supplemented with 1% Triton X-100 (Sigma-Aldrich, St. Louis, MO, USA) for 4 h. Absorbance was measured at 415 nm an Infinite M200 PRO microplate reader (Tecan Group Ltd., Männedorf, Switzerland). The concentration of 10Panx1 (200 µM) was selected based on its extensive use in the literature as a functional Panx1 inhibitor in myeloid and inflammatory cell types, including dendritic cells and other phagocytic populations. Under the experimental conditions used, no overt morphological alterations, loss of apparent cellular integrity, or reductions in cell density were observed during confocal imaging. In addition, early antigen uptake kinetics (30 min and 2 h) were comparable between treated and untreated cells, supporting the use of these concentrations for functional inhibition rather than nonspecific cytotoxic effects [42,43,44]. For each independent experiment, background values were determined using the “DC + B3Z without OVA” condition and subtracted from all experimental wells within the same plate. Data were then normalized relative to this baseline control prior to pooling across independent experiments.

### 4.8. Immunofluorescence Detection of Panx1 in BMDCs and MUTU1940 Cells

BMDCs and MUTU1940 cells were seeded onto glass coverslips (Thermo Fisher Scientific, Waltham, MA, USA) and allowed to adhere prior to staining. Cells were fixed with 2% paraformaldehyde (PFA; Sigma-Aldrich, St. Louis, MO, USA) and subsequently incubated with 200 mM NH4Cl (PFA; Sigma-Aldrich, St. Louis, MO, USA) to quench residual aldehyde groups. After washing with PBS, cells were permeabilized with 0.1% Triton X-100 for 10 min at room temperature. Between each step, cells were washed with PBS. Non-specific binding sites were blocked by incubation with immunofluorescence (IF) blocking medium for 30 min at 4 °C. Cells were then incubated with a rabbit anti-Panx1 primary antibody (Antibodies, A87666, Cambridge, UK) for 30 min at 4 °C, followed by PBS washes. BMDC samples were subsequently incubated with an Alexa Fluor 488-conjugated goat anti-rabbit IgG secondary antibody (Invitrogen, A32731, Thermo Fisher Scientific, Waltham, MA, USA), whereas MUTU1940 cells were incubated with an Alexa Fluor 647-conjugated goat anti-rabbit IgG secondary antibody (Invitrogen, A21244, Thermo Fisher Scientific, Waltham, MA, USA) for 30 min at 4 °C. After additional washes, nuclei were stained with DAPI (Thermo Fisher Scientific, Waltham, MA, USA) and samples were mounted for imaging. Fluorescence images were acquired using a Zeiss LSM 800 confocal microscope under identical acquisition settings for all experimental conditions. Negative-control samples were processed in parallel using the corresponding secondary antibody alone, omitting the primary antibody.

### 4.9. Statistical Analysis

Antigen redistribution and functional cross-presentation, as measured by B3Z activation assays, were analyzed using the non-parametric Mann–Whitney test. Statistical analyses were performed using GraphPad Prism software version 8.0.1 (GraphPad Software Inc., La Jolla, CA, USA). Data are presented as mean ± SEM, and differences were considered statistically significant at *p* < 0.05. One-tailed statistical tests were applied to functional cross-presentation assays based on a priori directional hypotheses, specifically predicting a reduction in cross-presentation following pharmacological inhibition of Panx1. In contrast, image-based colocalization metrics (Manders’ coefficients M1 and M2, and Pearson’s correlation coefficient) were analyzed using two-tailed tests, as changes in either direction were considered biologically informative and no directional assumption was imposed. Pairwise comparisons were restricted to pre-specified biologically relevant conditions defined before data analysis (e.g., untreated versus inhibitor-treated groups). Statistical analyses were therefore performed only to evaluate specific experimental hypotheses and not to compare all possible group combinations within each dataset. Consequently, no multiple-comparisons correction was applied.

## 5. Conclusions

In this study, we provide functional evidence supporting a role for Panx1 in intracellular antigen handling associated with antigen cross-presentation in dendritic cells. By combining quantitative confocal imaging with functional readouts of CD8^+^ T-cell activation, we show that pharmacological inhibition of Panx1 alters the intracellular redistribution of exogenous antigen and is accompanied by a reduction in MHC-I-restricted T-cell activation. These effects were consistently observed in GM-CSF/IL-4-derived inflammatory BMDC-like cells as well as in the cDC1-like MUTU1940 cell line, indicating that the contribution of Panx1 is not restricted to a single dendritic-cell model under the experimental conditions examined. Moreover, the lack of additive effects between Panx1 inhibition and P2X7 blockade, together with comparable responses in P2X7-deficient cells, suggests that Panx1-associated modulation of cross-presentation occurs largely independently of P2X7 signaling in this context. Importantly, our findings do not support the translocation of intact protein antigens through the Panx1 channel. Instead, they are consistent with a model in which Panx1 influences intracellular antigen redistribution and processing events associated with efficient cross-presentation. While this study does not establish direct genetic causality, it expands the current framework of antigen processing by identifying Panx1 as a modulatory component of intracellular antigen handling during cross-presentation. Together, these results highlight Panx1 as a previously underappreciated factor influencing antigen-processing events that culminate in CD8^+^ T-cell activation and provide a foundation for future studies employing genetic and in vivo approaches to further define its mechanistic role and physiological relevance.

## Data Availability

The original contributions presented in this study are included in the article/Supplementary Material. Further inquiries can be directed to the corresponding authors.

## Supplementary Materials

The following supporting information can be downloaded at: https://www.mdpi.com/article/10.3390/ijms27125559/s1.
