# Supplementary material for: Cross-Presentation and Activation of CD8+ T Cells: The Role of Pannexin-1 in Dendritic Cells
Source: Int J Mol Sci. 2026 Jun 19;27(12):5559. doi: 10.3390/ijms27125559 (PMC13299963; doi:10.3390/ijms27125559)
Supplement: Supplementary file 1 [file ijms-27-05559-s001.zip › ijms-4108473-supplementary.pdf]

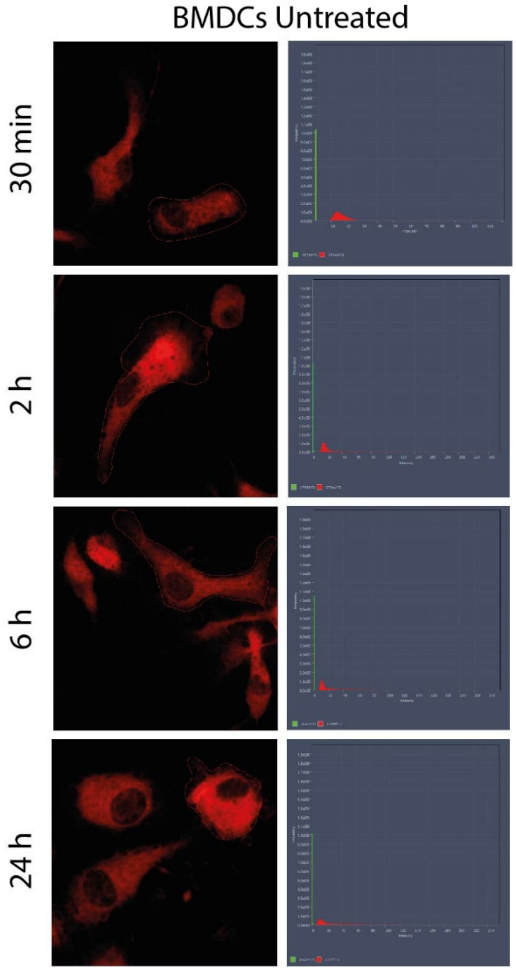

Supplementary Figure S1. Fluorescence intensity histograms used to define background and threshold settings for image analysis. Representative confocal images and corresponding fluorescence intensity histograms of BMDCs incubated under phagocytosis control conditions at 30 min, 2 h, 6 h, and 24 h. Histograms show minimal OVA–Alexa Fluor 488 signal, corresponding to background fluorescence levels. These conditions were used to establish fluorescence thresholds and background subtraction parameters applied consistently across all experimental groups. Images and histograms are representative of n = 6 independent experiments. Image acquisition and analysis were performed using ZEN software v3.13 (Zeiss)

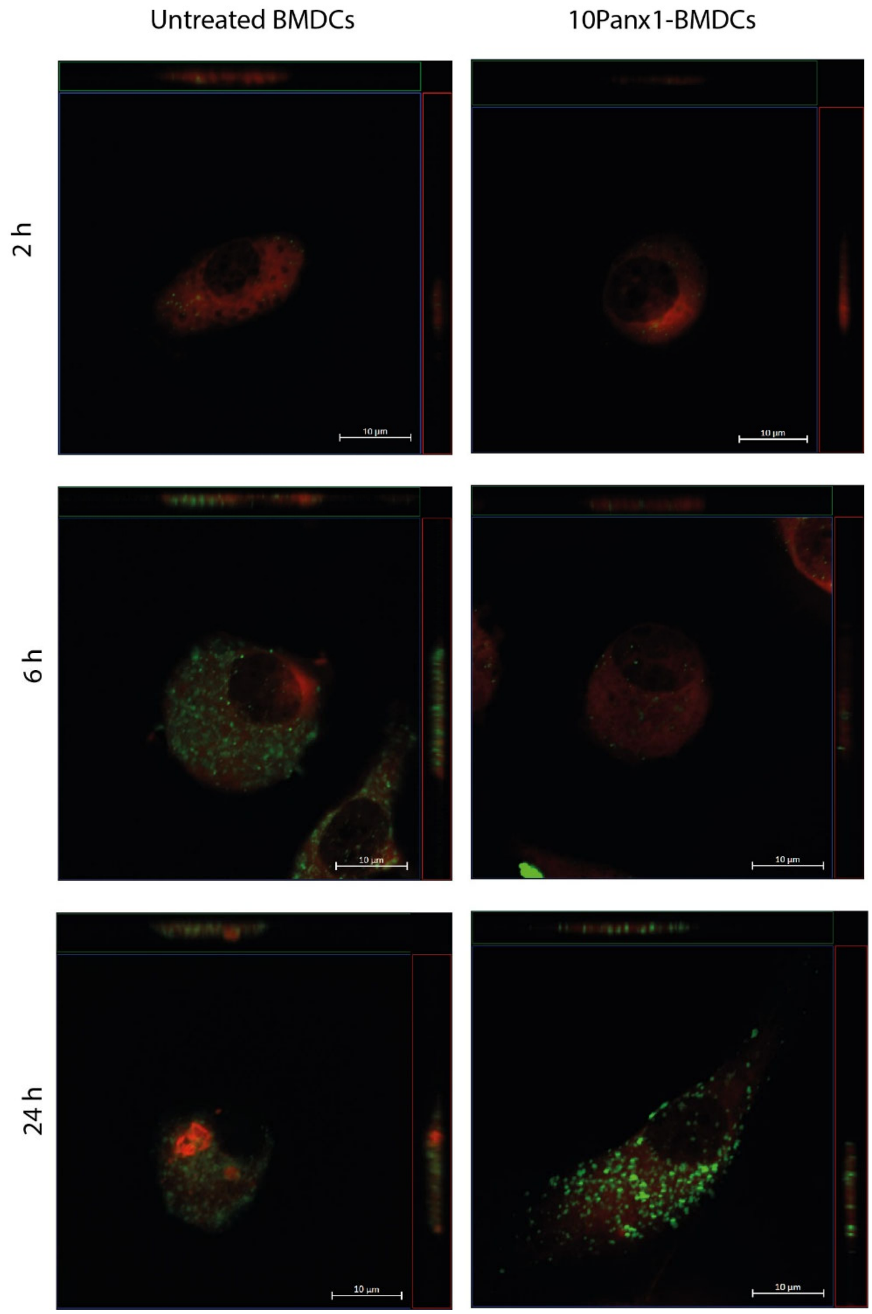

Supplementary Figure S2. Orthogonal views of OVA–Alexa Fluor 488 distribution in BMDCs. Representative orthogonal (XZ and YZ) views of confocal stacks from untreated BMDCs and BMDCs treated with the Panx1 inhibitory peptide (10Panx1) at 2 h, 6 h, and 24 h after incubation with OVA–Alexa Fluor 488. Orthogonal projections were generated from the same confocal datasets shown in Figure 1 to visualize the spatial distribution of the antigen within the cellular volume. Cell boundaries were defined using CellTracker™ Red CMTPX, and orthogonal planes are indicated by colored reference lines in the main panels. Images are representative of $n = 6$ independent experiments. Scale bar: 10 µm. Image acquisition and processing were performed using ZEN software v3.13 (Zeiss).

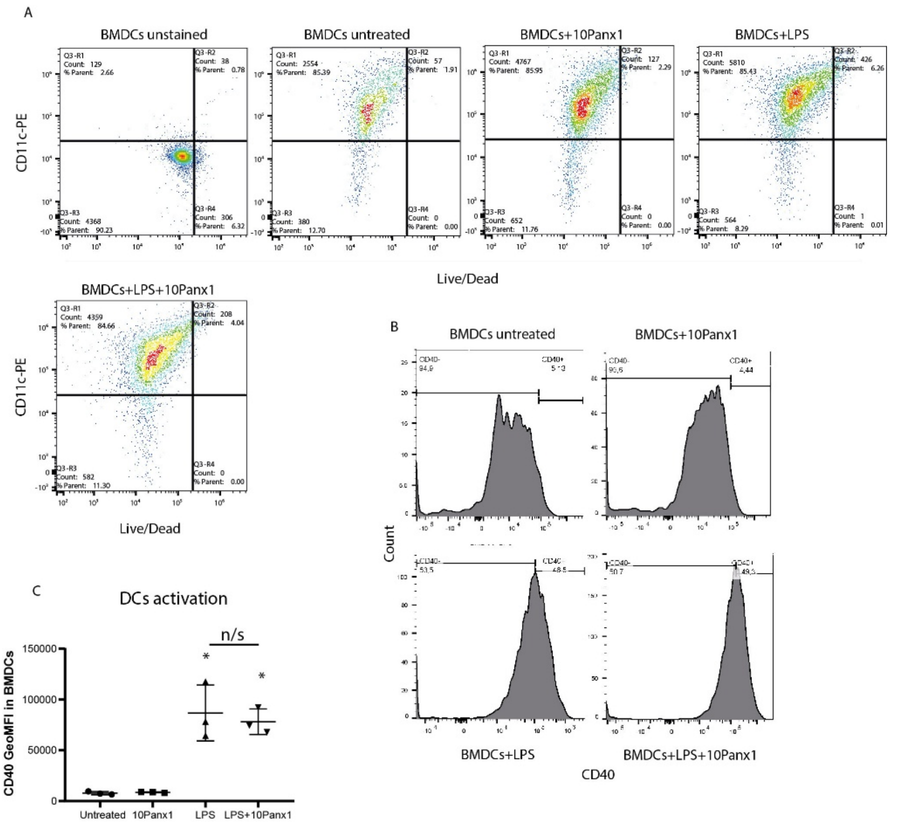

Supplementary Figure S3. PANX1 inhibition does not significantly affect BMDC viability or LPS-induced activation. (A) Representative flow-cytometry dot plots showing Live/Dead staining in untreated BMDCs, BMDCs treated with 10Panx1 (200 μM), BMDCs stimulated with LPS, and BMDCs stimulated with LPS in the presence of 10Panx1. Comparable frequencies of viable cells were observed across all conditions. (B) Representative histograms showing CD40 expression in untreated BMDCs, BMDCs treated with 10Panx1, BMDCs stimulated with LPS, and BMDCs stimulated with LPS plus 10Panx1. LPS induced robust upregulation of CD40 expression, whereas treatment with 10Panx1 alone did not significantly alter basal CD40 levels. (C) Quantification of CD40 geometric mean fluorescence intensity (GeoMFI) in BMDCs. LPS significantly increased CD40 expression compared with untreated controls, while no significant differences were observed between LPS-stimulated cells in the presence or absence of 10Panx1. Data are presented as mean ± SEM from three independent experiments. Statistical significance was evaluated using the Mann–Whitney test. *$p < 0.05$; n/s, not significant.

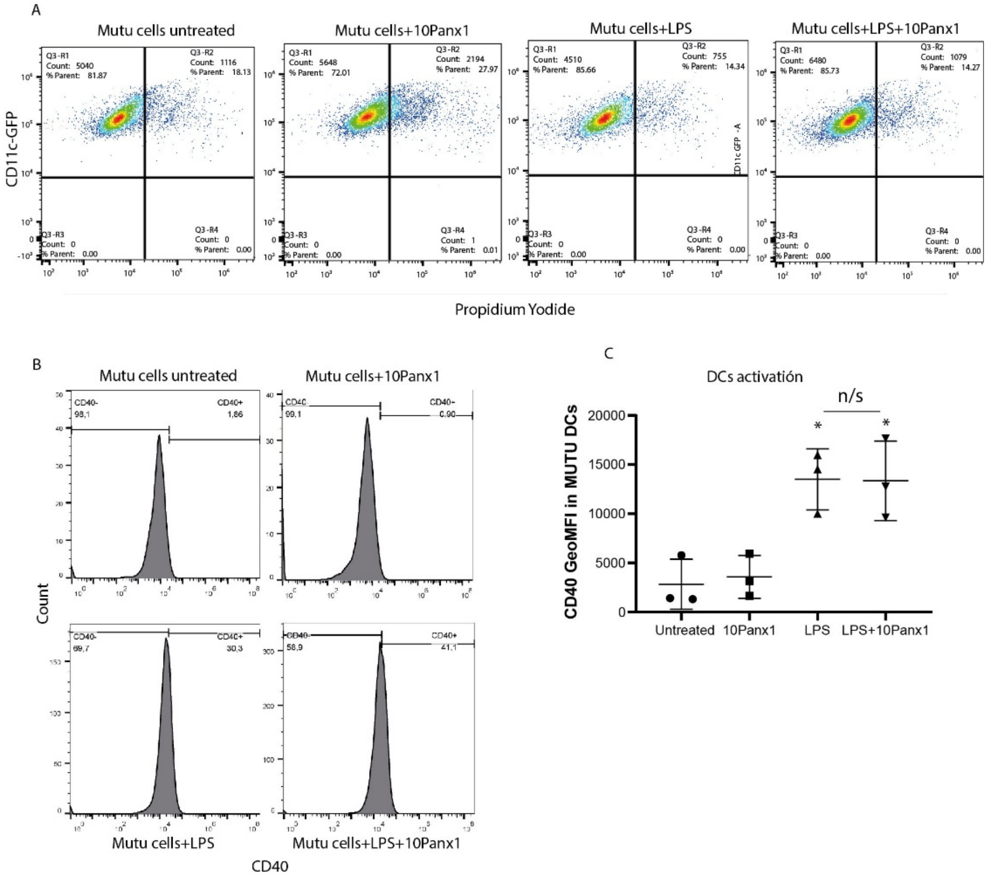

Supplementary Figure S4. PANX1 inhibition does not significantly affect MUTU1940 cell viability or LPS-induced activation. (A) Representative flow-cytometry dot plots showing propidium iodide (PI) staining in untreated MUTU1940 cells, cells treated with 10Panx1 (200 µM), cells stimulated with LPS, and cells stimulated with LPS in the presence of 10Panx1. Comparable frequencies of PI-negative viable cells were observed across all conditions. (B) Representative histograms showing CD40 expression in untreated MUTU1940 cells, cells treated with 10Panx1, cells stimulated with LPS, and cells stimulated with LPS plus 10Panx1. LPS induced marked upregulation of CD40 expression, whereas treatment with 10Panx1 alone did not significantly modify basal CD40 levels. (C) Quantification of CD40 geometric mean fluorescence intensity (GeoMFI) in MUTU1940 cells. LPS significantly increased CD40 expression compared with untreated controls, while no significant differences were detected between LPS-stimulated cells in the presence or absence of 10Panx1. Data are presented as mean ± SEM from three independent experiments. Statistical significance was evaluated using the Mann–Whitney test. *$p < 0.05$; n/s, not significant**.**
